# Community pharmacists’ routine provision of drug-related problem-reduction services

**DOI:** 10.1371/journal.pone.0267379

**Published:** 2022-05-04

**Authors:** Ghaith M. Al-Taani, Nehad M. Ayoub

**Affiliations:** 1 Department of Clinical Pharmacy and Pharmacy Practice, Faculty of Pharmacy, Yarmouk University, Irbid, Jordan; 2 Department of Clinical Pharmacy, Faculty of Pharmacy, Jordan University of Science and Technology (JUST), Irbid, Jordan; Jouf University, Kingdom of Saudi Arabia, SAUDI ARABIA

## Abstract

**Objectives:**

The present study aimed to assess the degree of the provision of services for drug-related problems (DRPs) and the factors affecting provision within the community pharmacy setting in Irbid, a large city in Northern Jordan.

**Methods:**

A cross-sectional survey was developed and administered to community pharmacists in Irbid, Jordan during the period from January to May 2017. The survey is composed of background and practice characteristics, services provided routinely by the community pharmacists to address DRPs, and barriers and facilitators for DRP-reduction services. A summated score quantifying the degree of DRP-reduction service provision was calculated, which included overall scores and scores for the different scales and domains. Statistical analysis included descriptive statistics and a multivariate linear regression model for factors associated with the high provision of DRP-reduction service.

**Results:**

Two hundred community pharmacists out of 210 pharmacists approached completed the surveys yielding a response rate of 95.2%. The most frequent DRPs encountered within the routine practice in the community pharmacy were economic aspects (76.0%). The mean total score relating to different DRP-reduction services was 32.9 (58.8%) out of 56 as the maximum possible score. It was estimated that 28.2% of the responding pharmacists provided the service overall (scored more than 50% of the scale). For the assessment, intervention, and referral dimensions, similar percentages of providers of the services were achieved: 59.7%, 61.9%, and 49.0%, respectively. Lower rates of providers were achieved on the documentation scale (12.9%). The lack of recognition of the pharmacist role by physicians was the most commonly reported barrier to effective DRP-reduction services among community pharmacists (78.9%). The ability to receive external guidance was indicated by the majority of surveyed pharmacists (94.5%) as a potential facilitator to DRP-reduction services in this study. Predictors associated with high total scores were the presence of medical records for the patients in the pharmacy, patients contact the pharmacy using email, a high satisfaction in professional relationships with physicians, and pharmacists’ age.

**Conclusion:**

Even though community pharmacists in this study have been shown to deliver certain activities to address DRPs to a high degree, the overall rate of DRPs services was suboptimal. Community pharmacists reported several barriers that should be taken into consideration to facilitate the role of community pharmacists in providing adequate DRP reduction services to patients.

## Introduction

Pharmaceutical care is a growing pharmacy practice delivered to patients in collaboration with healthcare providers [1]. With the increased complexity of pharmacotherapy, issues relating to medicine use could arise. The cornerstone for the practice of pharmaceutical care is to support and provide advice on prescribing and managing medication use issues both present and anticipated; these issues are usually referred to as drug-related problems (DRPs). DRPs are defined as “*events or circumstances involving drug therapy that actually or potentially interferes with desired health outcomes*” [2].

Community pharmacists are the most accessible healthcare providers for the general public, and they represent a cornerstone sector of the healthcare system [3]. With the recent advancements in the profession of pharmacy, the role of community pharmacists had transitioned from a classical product-focused approach to a patient-oriented one [3, 4]. In such settings, the pharmacist-provided services could expand to take important roles in disease prevention, screening, monitoring of drug therapy, and patient adherence to medications [5]. Furthermore, pharmacy services proved to have a significant impact on improving patient outcomes and healthcare in the developed countries [4, 6]. Using survey methodology, several studies have assessed the provision of direct patient care pharmacist services using data collected from a large number of pharmacies. Using this approach, many studies have assessed the degree of provision of pharmaceutical care in community pharmacies [7–15]. The provision of pharmaceutical care was assessed using the Behavioral Pharmaceutical Care Scale (BPCS) and other specifically developed instruments [8, 10, 11, 14, 15]. These studies scrutinized the pharmaceutical care provision, in all its dimensions, through routine patient care, i.e., not within research studies, yielding similar trends across different settings and scales, that highlighted the issue of limited provision of pharmaceutical care services within the community pharmacy setting globally [7–15].

Rossing et al. (2003) distributed a mailed questionnaire that assessed the provision of DRP services in the community pharmacy setting in Denmark. They found that DRPs were detected in approximately 50% of all patients despite a low provision of services related to setting goals and documentation. Their instrument was short, consisting of fewer than 5 items with minimal background information; thus, it did not provide clear information about the provision of DRP services [11]. A large European study that assessed the provision of pharmaceutical care in 13 countries found that such provision was limited. The study concluded a limited provision of pharmaceutical care in a large European sample of community pharmacies [7]. Locally, AbuRuz et al. (2012) reported that the overall provision of pharmaceutical care was limited within community pharmacies in Jordan, despite the high provision of certain services, such as providing advice [15]. Although these studies [7, 15] provided insights into the direct patient care activities within community pharmacy settings, their findings were preliminary and did not provide a clear and detailed description of DRP services within the community pharmacy setting.

Despite the progress in pharmacy practice, the utilization of pharmacy services in developing countries is limited compared to developed countries [4]. In Jordan, there are 3500 community pharmacies distributed across the country in 2020 and most of the pharmacy graduates work as community pharmacists [16]. Nevertheless, patient-oriented pharmacy practice is not sufficiently established mainly as a result of inadequate services offered by community pharmacists in Jordan [16]. In a study by Mukattash et al., the majority of the public in Jordan indicated that dispensing of medications and patient counseling are the most important roles of community pharmacists [17]. Recent studies exploring DRPs have been also carried out in Jordan. They have typically focused on pharmaceutical care research assessing the impact of clinical pharmacy interventions within the outpatient and hospital settings [18, 19], with other studies assessing other issues related to DRPs, such as their prevalence [20–22]. There are few studies conducted globally that assessed DRP reduction services provision specifically and most of the studies that addressed DRP reduction services provision in Jordan were part of research studies, i.e., not routine practice. Thus, the present study aimed to assess the degree of provision of DRP-reduction services in routine clinical care by community pharmacists working in community pharmacies in Irbid, a large city in Northern Jordan.

## Materials and methods

A cross-sectional descriptive survey was conducted in which a hand-delivered questionnaire was distributed to a convenience sample of licensed pharmacists in community pharmacies during the period January–May 2017.

### Data collection and study procedure

A structured questionnaire assessing the degree of provision of DRP services within community pharmacies was developed. The content of the questionnaire was guided by published research in this area [7, 8] and was reviewed by faculty members within the research area for face and content validity. The content validity index was calculated based on input from 8 faculty members with Ph.D. or MSc degrees in Pharmacy with clinical expertise. The results revealed an average content validity index of 72.1% for the total scale and 75% for all subscales apart from the documentation scale which was 63.5%. Convergent validity was confirmed by a high correlation of each subscale with the total scale which is statistically significant and Pearson coefficient above 0.208. The Pearson correlation was as 0.794, 0.896, 0.679, and 0.705 for the assessment, intervention, referral, and documentation subscales, respectively. Thus, the content validity index provides a basis for the validity of the instrument. The questionnaire was then distributed to a pilot group of community pharmacists (n = 10). Minor changes to the content and layout of the questionnaire were made based on responses from the pilot group to improve the clarity of the questionnaire. Data from the pilot group were excluded from the final analysis.

The scale’s reliability analysis revealed a Cronbach’s alpha value of 0.807, when the subscales were summed Cronbach’s alpha value was 0.775, and each section Cronbach’s alpha was considerably lower ranging from 0.482–0.633. The questionnaire was developed in Arabic and was not subjected to translation from another language.

A list of community pharmacies in Irbid was used as a sampling frame which was obtained from the Ministry of Health. The distribution of the survey included community pharmacists in both urban and rural areas in Irbid city. Non-random convenience sampling approach was used to recruit pharmacists to this study. Pharmacists who have a bachelor’s degree in pharmacy (BPharm), a Doctor of Pharmacy (PharmD) degree, or a higher educational degree were eligible to participate in the study.

The questionnaire was distributed by a trained research assistant via a face-to-face approach in paper format. The research assistant approached pharmacists in their community pharmacies and explained the study goals and procedures and that participation is voluntary. Pharmacists who agreed to participate in the study were requested to provide written informed consent. Those who refused to participate or did not provide informed consent were not eligible to complete the survey. To ensure the confidentiality of responses, the data collected was anonymous. Community pharmacists who participated in the study completed the survey with the presence of the research assistant to clarify any unclear items in the survey; such an approach assures consistency in answering issues that might be raised by participants during data collection. The time taken to complete the survey was about 10–15 minutes. After administration, the research assistant collected back the survey from the participants.

The target sample size was 184 responses, based on a 5% margin of error and a 95% confidence level, from a sampling frame of 350 community pharmacies in Irbid. Ethical approval was obtained from Institutional Review Board at King Abdullah University Hospital, Irbid, Jordan (Reference No.: 71/136/2020). The information about the study and the informed consent form was embedded as the first part of the questionnaire.

### Instrument

The questionnaire comprised three main sections: (1) Demographic information of community pharmacists and practice settings of the pharmacies; (2) Items related to DRPs in terms of types, most commonly implicated drugs, and application in direct patient care; and (3) Pharmacists’ views regarding barriers to and facilitators of DRP services within community pharmacy settings.

Demographic and practice characteristics included a total of 9 items to obtain information on age, gender, education, years of experience in community pharmacy, work hours per week, the type of community pharmacy, the total number of pharmacists in the pharmacy, the presence of counseling area, and the workload as described by community pharmacists.

The second part of the questionnaire describes items related to the DRPs. These included the source of knowledge of DRPs (one item), overall provision of DRP services (one item), most common DRPs encountered in routine practice (one item), most common drug group implicated with DRPs (one item), and the provision of different behaviors related to DRPs (14 items). The present study proposed that the behaviors fell within four subscales: assessment, intervention, referral, and documentation, as a classification scheme. Individual items asked about how often pharmacists perform DRP-related activities (i.e., always provided, most of the time provided, some of the time provided, and never provided, with corresponding weights being 4, 3, 2, and 1, respectively). These weights were summed to calculate a subscale score and a total score of the instrument.

The third part of the questionnaire describes perceived barriers to and facilitators toward the provision of DRP-reduction services among pharmacists in community pharmacy settings. Eleven potential barriers were listed including limited time, increased workload, lack of training, lacking laws defining professional pharmacist roles regarding DRP, no guidelines for the role of the pharmacist, pharmacist role is not accepted by physicians, pharmacist role is not accepted by patients, lack of confidence, lack of incentives, lack of tools to perform the service, and inappropriate layout of the pharmacy. A total of 6 facilitators were listed. These included a good relationship with physicians, an incentive for the service, appropriate pharmacy layout, workforce sufficient and appropriately trained, ability to receive external guidance, and training of pharmacist.

### Outcome measures

The proposed subscales for the DRP questionnaire included, as mentioned earlier, are behaviors associated with the provision of services to address DRPs. The first subscale was the assessment subscale that identify health problems and needs and how they would be addressed related to DRPs, which was surveyed via the items review records to assess for DRPs, asking patients regarding DRPs, set goal for DPRs, and assess for DRPs. The minimum possible score for this subscale was 4 and the maximum possible score was 16. The next subscale is the intervention subscale that is action to improve the patient outcome, which was surveyed via the items propose interventions, develop an intervention in the pharmacy, plan to resolve DRPs, and follow-up of the patient. The minimum possible score for this subscale was 4 and the maximum possible score was 16. Another subscale was the referral subscale which involves directing the patient to another healthcare professional, that was surveyed detailing the referral to a medical doctor, other healthcare professionals, and/or pharmacist. The minimum possible score for this subscale was 3 and the maximum possible score was 12. The documentation subscale was related to recording health information, either paper-based or electronic that another healthcare professional can interpret and be surveyed in the present study as documentation, document intervention, and document plan. The minimum possible score for this subscale was 3 and the maximum possible score was 12. The total score that included the sum of all subscales was calculated and it can take any value between the minimum possible score for the scale (14) and the maximum possible score for the scale (56). To devise a more informative approach, the midpoint of the scale has been selected to dichotomize the responses and further categorize the respondents as providers and non-providers of the behavior in question.

### Data analysis

Data were entered to SPSS v19.0 for detailed statistical analyses. Descriptive statistics were used and data were presented as frequency and percentages (n, %) for categorical data, or the mean for continuous variables. The scores for the different scales of the questionnaire were summed. An evaluation of any significant relationships between DRP service provision (and their subtypes) with the demographic information was carried out. An assessment of the risk factors associated with total scores using linear regression analysis. Important candidate variables were identified via an initial automatic linear regression analysis (using SPSS software), and these variables were then included in a “forward” linear regression model, retaining only independent predictors with a statistically significant contribution to the outcome measure. Statistical significance was set at P<0.05.

## Results

Two hundred surveys were completed, representing approximately two-thirds of community pharmacies in Irbid. This response was obtained from a total of 210 participants approached in their community pharmacies, yielding a response rate of 95.2%.

Approximately 90% of the community pharmacist respondents were females. The results revealed that 82.2% of respondents were 30 years or younger. The majority of respondents (78.4%) held BPharm. Regarding pharmacists’ experience, 47.7% had two years of experience or less in community pharmacy settings. The majority (82.4%) of pharmacists work full-time in the community pharmacy (41–50 hours per week). Approximately two-thirds (64.0%) of the community pharmacies were independent, whereas 36% of the pharmacies were chain pharmacies. It was most common (46.7%) for two pharmacists to work in the pharmacy. A private area for patient counseling was present in 73.0% of pharmacies. Responding pharmacists expressed that their workloads were too heavy in 9.0% of cases, average in 76.0% of cases, low in 12.0% of cases, and very low in 3.0% of cases. Table 1 summarizes the characteristics of the responding pharmacists and their community pharmacy settings.

**Table 1:** Demographic and practice characteristics of the community pharmacists (N = 200).

Variable		n	%
**Demographic characteristics**			
Gender	Male	21	10.5
	Female	179	89.5
Age, years	≤ 25	81	49.7
	26–28	45	27.6
	29–30	8	4.9
	31–40	15	9.8
	41–50	11	6.7
	51–60	1	0.6
	> 60	1	0.6
Education	BPharm	156	78.4
	PharmD	24	12.1
	Graduate	6	3.0
	Other	13	6.5
**Practice settings and characteristics**			
Experience as a community pharmacist, years	≤ 2	93	47.7
	3–5	55	28.2
	6–10	25	12.8
	11–20	15	7.7
	> 20	7	3.6
Weekly work hours	2–20	8	4.0
	21–40	16	8.0
	41–50	164	82.4
	≥ 51	11	5.5
Type of community pharmacy	Independent	128	64.0
	Chain	72	36.0
Total number of pharmacists in the community pharmacy	1	53	26.6
	2	93	46.7
	3	33	16.6
	4	15	7.5
	5	4	2.0
Counseling area	Yes	146	73.0
	No	54	27.0
Workload	Too much	18	9.0
	Average	152	76.0
	Low	24	12.0
	Very low	6	3.0

BPharm, Bachelor’s in pharmacy; PharmD, Doctor of pharmacy

In relation to items related to DRPs as reported in Table 2, respondents’ source of knowledge about DRPs came mostly from their university education (78.5%) and the workplace (19.0%). When asked about the overall provision, the respondents indicated that DRP-reduction services are always provided (18.5%), often provided (43.5%), sometimes provided (31.5%), rarely provided (4.5%), and never provided (2.0%) during the routine work practice.

**Table 2:** Overall provision of DRP service, DRPs encountered, and drugs commonly associated with DRP services among community pharmacists (N = 200).

Variable		n	%
Source of DRP knowledge	University	153	78.5
	Postgraduate study	2	1.0
	Workplace	37	19.0
	Other	3	1.5
How often do you provide DRP-reduction services during your routine practice?	Always	37	18.5
	Often	87	43.5
	Sometimes	63	31.5
	Rarely	9	4.5
	Never	4	2.0
DRPs commonly encountered in routine community pharmacy practice	Therapeutic failure	55	27.5
	Adverse drug effect	86	43.0
	Problem in patient’s knowledge about drug or disease	103	51.5
	Adherence problems (drug or lifestyle)	126	63.0
	Efficacy problems	25	12.5
	Duration of treatment	103	51.5
	Economic aspects	152	76.0
	Non-indicated drugs	45	22.5
	Untreated condition	37	18.5
	Contraindicated drug	53	26.5
	Addiction use	76	38.0
	Drug interaction	68	34.2
	Duplication	83	41.5
Drugs commonly associated with DRPs as identified by the pharmacist in routine community pharmacy practice	Gastrointestinal drugs	20	10.1
	Central nervous system drugs	27	13.6
	Respiratory system drugs	5	2.5
	Genitourinary drugs	7	3.5
	Antibiotics	82	41.4
	Endocrine drugs	3	1.5
	ENT drugs	4	2.0
	Musculoskeletal drugs	1	0.5
	Cardiovascular drugs	20	10.1
	Ophthalmic drugs	4	2.0
	Blood and nutrition drugs	1	0.5
	Dermatological drugs	24	12.1

ENT, Ear-Nose-Throat.

Many types of DRPs are commonly encountered/addressed within the routine practice in the community pharmacy. The most common DRPs encountered are economic aspects (76.0%), drug or lifestyle adherence problems (63.0%), duration of treatment, and problems regarding the knowledge of patients about a drug or the disease (each accounts for 51.5%). Alternatively, efficacy problems were the least encountered type of DRPs as indicated by the surveyed pharmacists in this analysis (12.5%) (Table 2). Regarding drugs commonly associated with DRPs that respondents routinely identify, antibiotics were reported by 41.4% of respondents, and central nervous system drugs were reported by 13.6% of respondents (Table 2).

Data presented in Table 3 report the mean scores for the total of the scale (total score) and different subscales and the provision of the specific tasks related to DRP-reduction services together with the percentage of the mean score from the total possible score and percentage of responders who scored more than 50% of the scale (i.e., provide service). The mean total score was 32.9 out of 56, the maximum possible score, representing 58.8% of the maximum possible score. It was estimated that 28.2% of the respondents provided the service overall i.e., scored more than 50% of the scale. The assessment, intervention, and referral dimensions showed similar percentages of providers of the services namely, 59.7%, 61.9%, and 49.0%, respectively. Meanwhile, a lower percentage of providers were achieved on the documentation scale, which was 12.9% of the total achievable score.

**Table 3:** Total and dimension scores and provision of various DRP service-related activities among community pharmacists (N = 200).

**Total and dimension scores**					
	Mean score		Percentage of the mean score from the total possible score		Percentage of responders who scored more than 50% of the scale (i.e., provide service)
Total score	32.9		58.8		28.2
Assessment scale	10.2		63.8		59.7
Intervention scale	10.4		65.0		61.9
Referral scale	7.6		63.3		49.0
Documentation scale	4.9		40.8		12.9
**Provision of various DRP service-related activities**					
	Service	Always provided n (%)	Most of the time provided n (%)	Some of the time provided n (%)	Never provided n (%)
Assessment scale	Review record to assess for DRPs	7 (3.5)	19 (13.0)	31 (15.5)	143 (71.5)
	Ask patient regarding DRPs	90 (45.5)	64 (32.3)	29 (14.6)	15 (7.6)
	Set goal for DRPs	97 (49.2)	63 (32.0)	28 (14.2)	9 (4.6)
	Assess for DRPs	38 (19.1)	47 (23.6)	58 (29.1)	56 (28.1)
Intervention scale	Propose alternative	34 (17.2)	64 (32.3)	66 (33.3)	34 (17.2)
	Develop intervention in pharmacy	105 (53.0)	65 (32.8)	19 (9.6)	9 (4.5)
	Plan resolve	27 (13.5)	36 (18.2)	40 (20.2)	95 (48.0)
	Follow up	50 (25.4)	55 (27.9)	49 (24.9)	43 (21.8)
Referral scale	Doctor	94 (47.2)	67 (33.7)	32 (16.1)	6 (3.0)
	Other HCPs	43 (21.7)	39 (19.7)	38 (19.2)	78 (39.4)
	Pharmacist	28 (14.1)	37 (18.6)	58 (29.1)	76 (38.2)
Documentation scale	Documentation	9 (4.5)	20 (10.1)	28 (14.1)	142 (71.4)
	Document intervention	24 (12.2)	21 (10.7)	29 (14.8)	122 (62.2)
	Document plan	16 (8.1)	23 (11.6)	48 (24.2)	111 (56.1)

On the assessment scale, only a few (16.5%) responding pharmacists always or most of the time reviewed records to assess for DRPs and 77.8% always or most of the time asked the patients questions to assess DRPs (Table 3). The pharmacists provided interventions to address DRPs, e.g., 49.5% always or most of the time proposed alternatives and 85.8% always or most of the time provided interventions in the pharmacy. A total of 80.9% of the responding pharmacists always or most of the time referred patients to physicians, 41.4% always or most of the time referred patients to other healthcare professionals, and 32.7% always or most of the time referred patients to another pharmacist. Regarding the documentation subscale, 14.6% of the responding pharmacists always or most of the time documented information related to DRPs (Table 3).

Table 4 describes providers of DRP services and non-providers of DRP services and predictors associated with the total scale score. Providers of DRP services were defined as those who scored in the top 10% of total scores, which was within the score range of 44 to 56; they constituted 10.6% of the total sample. Non-providers of DRP services scored in the bottom 10% of total scores, with scores ranging from 17 to 23; they accounted for 9.6% of the total sample. Statistically significant predictors associated with total scores were the presence of medical records for patients in the pharmacy, patients contacting the pharmacy using email, high satisfaction in professional relationships with physicians, and the age of the pharmacist. The R^2^ for the model equaled 17.7%.

**Table 4:** Providers and non-providers of DRP services and predictors associated with total scale score among community pharmacists (N = 200).

**Providers and non-providers of DRP services**					
Item	Definition		Score range		%
Non-providers of DRP services	bottom 10% of total score		17–23		9.6%
Providers of DRP services	top 10% of total score		44–56			10.6%
**Predictors associated with total scale score**					
Predictor	**Unstandardized Coefficients**		**95% C I for B**		**P value**
	B	Std. Error	Lower Bound	Upper Bound	
(Constant)	35.883	2.206	31.564	40.216	
Presence of medical record	3.709	1.306	1.127	6.291	0.005
Patient use of pharmacy email	4.465	1.599	1.304	7.626	0.006
Satisfaction with relationship with doctors	3.507	1.239	1.059	5.955	0.005
Age	-0.179	0.073	-0.323	-0.035	0.015

Table 5 summarizes the barriers and facilitators for the provision of DRP-reduction services from the view of responding pharmacists. The top barriers were the pharmacist’s action not being accepted by physicians (78.9%) and the lack of laws defining these new professional roles (63.6%). Many facilitators for DRP-reduction services were highlighted by a large number of responding pharmacists. For example, 94.5% of respondents identified the ability to receive external guidance as a facilitator, 92.0% identified the pharmacist’s training, and 91.5% identified the need for a sufficient number of trained workforce.

**Table 5:** Barriers and facilitators identified for provision of DRP-reduction services among community pharmacists (N = 200).

Variable		Agree or strongly agree	
		n	%
Barrier	Limited time	108	54.0
	Possibility of increased workload	104	52.5
	Lack of training	99	50.0
	No laws defining these professional roles	126	63.6
	No guidelines for the role of the pharmacist	121	61.1
	Pharmacist role is not accepted by physicians	157	78.9
	Pharmacist role is not accepted by patients	108	54.3
	Lack of confidence	35	17.6
	Lack of incentives	81	40.9
	Lack of tools to perform the service	92	46.2
	Layout of the pharmacy is inappropriate	54	27.0
Facilitator	Good relationship with physicians	181	91.0
	Incentive for the service	143	71.5
	Appropriate pharmacy layout	172	86.0
	Workforce sufficient and appropriately trained	183	91.5
	Ability to receive external guidance	189	94.5
	Training of pharmacist	183	92.0

## Discussion

The efficacy of pharmacy services to address DRPs has been traditionally documented in research studies (e.g., randomized controlled trials); however, the determination of the degree of provision of the day-to-day DRP-reduction services is important [23]. The present study quantified the degree of provision of DRP-reduction services by community pharmacists surveyed in a large city in Northern Jordan (Irbid) and found a limited provision of services to address DRPs by community pharmacists, which was quantified by 58.8% of the maximum possible score, according to the self-reported scale. Pharmacists assessed for DRPs, provided interventions, and referred patients, where appropriate; however, targets for continuous improvement were noted in relation to documentation, among other items.

The overwhelming majority (90%) of respondents were females. More females than males study pharmacy, and the professional body in Jordan, Jordan Pharmacists Association, includes more females than males. The majority of respondent pharmacists work full time in the pharmacy and earned a BPharm degree, which represents the standard legal requirement for staffing in pharmacy. The majority (82.2%) of respondents were 30 years or younger, which means that the study sample is somewhat young; younger professionals are more likely to implement cognitive professional services and keep pace with newer developments in professional practice [24]. In the present study, younger pharmacists were independent predictors of higher provision of DRP-reduction services.

Two-thirds of pharmacies where the pharmacists were surveyed were independent, while the remaining pharmacies were chain pharmacies. A similar survey in North Carolina reported that independent pharmacies are more likely to provide enhanced services than chain pharmacies, these enhanced services include services other than dispensing and advice on the selection of nonprescription drugs, such as medication therapy management, disease screening, and smoking cessation services [25, 26]. A national survey in the USA found that independent pharmacies dispensed more prescriptions than chain pharmacies [27], as the pharmacists dispense more prescriptions it is envisioned that there would be a higher workload that impedes the provision of DRP-reduction services or might be a facilitator for such services by increased expertise and experience when dealing with many prescriptions, however, the differential prescription volume across pharmacy type might be a special feature in the community pharmacy setting in the USA. Most community pharmacies had a total of two working pharmacists (46.7%), which allowed for a room for workspace interaction, which might facilitate the provision of patient-centered services [7, 26]. Furthermore, 76.0% of respondents in our study reported that the workload in their pharmacies was average, which can help deliver enhanced services, as lack of time is anticipated to decrease the provision of enhanced services [28, 29].

The role of the community pharmacist is shifting from dispensing services to providing more holistic, patient-centered services, and the impact of the pharmacist role in such innovative initiatives has been documented in research studies internationally [30]. Yet, counseling and dispensing remain key services from community pharmacies internationally [31].

In relation to the overall provision of DRP-reduction services, 18.5% of respondents always delivered them while 75.0% of respondents often and sometimes provided them. The role of pharmacists is becoming more diverse, with two competing focuses, the enhanced patient care services and inventory control [32], and the present finding supports high overall delivery of DRP-reduction services. However, such high delivery rates should be interpreted with caution as multiple studies have highlighted the high level of DRPs in different settings in Jordan [21, 22], and such high provision rates could be a result of respondents over-reporting “good behavior”. Such phenomena represent social desirability bias [33]. However, in a Jordanian study within community pharmacy settings, 76% of the respondents agreed to the idea of providing pharmaceutical care interventions for free and would view that provision of such services can attract business to the pharmacy [34].

The mean total score, representing a quantitative measure of the routine provision of DRP-reduction services by community pharmacists, equaled 32.9, representing 58.8% of the total achievable score. This indicates the limited routine provision of DRP-reduction services by the community pharmacists surveyed. Such limited provision is consistent with what has been published, as other studies have been carried out to examine a similar theme in European community pharmacies internationally [7, 35]. A study in the USA reported that 31% of responding community pharmacists provided medication therapy management [29] whereas, in Australia, 88% of community pharmacists reported providing one or more enhanced pharmacy services [24]. Patient-centered services were found to be provided at a modest rate in a number of international studies [29, 36], however, in the UK, 87% of community pharmacies provided enhanced services, and medication review services are provided to a high extent that is even increasing [37]. The provision of pharmaceutical care was limited by community pharmacists in a number of international studies in Denmark, Spain, China, Jordan, and the USA [10–15].

The overall limited provision of DRP-reduction services identified in this study is consistent with international studies carried out in European community pharmacy settings, which found a limited provision of pharmaceutical care services [7, 35]; however, some services were delivered to a slightly higher degree, such as assessments, interventions, and referrals. Specific tasks related to DRP-reduction services were delivered to a high extent, including the assessment of DRPs by inquiring patients, setting goals for DRP issues, providing interventions in the pharmacy, and referring patients to a medical doctor. Similarly, a survey carried out in Jordan quantifying the degree of provision of pharmaceutical care services reported that 96.8% of community pharmacy respondents assessed for DRPs by asking questions [34]. In a 2004 national survey in the USA, health promotion was provided by more than 80% of the respondents, whereas more enhanced services were provided at a lower rate [38]. At that time, there has been not much appetite for enhanced services, which would not be the case now with newer legislation and practice frameworks.

On the other hand, some specific tasks related to DRP-reduction services were delivered by a few pharmacists, including the review of records for DRP identification, the carrying out of plans to address DRPs, patient referrals to other pharmacists, documentation of DRPs, documentation of interventions to resolve DRPs, and documentation of the plan to resolve DRPs. Such low provision is not expected to be the case in developed countries, where the pharmacy plays a more diverse role [24]. In European countries, considering community pharmacy in relation to the provision of pharmaceutical care services, a low provision of services related to patient assessment and the implementation of therapeutic objective and monitoring plans has been reported [7, 35].

The characteristics of community pharmacists associated with the provision of DRP-reduction services, accounted for via total score, were the presence of patients’ medical records in the pharmacy, the contacting of patients using email, community pharmacists’ high satisfaction in their relationships with physicians, and community pharmacists’ age. These are important independent factors associated with the higher provision of DRP-reduction services, which provides a framework to what we can be done to enhance DRP-reduction services provision. Similar to the present study findings regarding younger age being a predictor of enhanced DRP-reduction service provision in British Columbia, the provision of enhanced services occurred among those pharmacists with appropriate attitudes and recent graduation dates [39]. In an Australian survey, one facilitator of enhanced pharmacy services within the community pharmacy was “access to patient notes” [24]. However, clinical information about patients was available in fewer than 50% of European community pharmacies via shared databases with other healthcare institutions [35]. It is well known that in the community pharmacy settings in Jordan, such shared databases are not present. In this study, a similar trend was noted concerning the presence of patient’s medical records in the pharmacy as a predictor for enhanced DRP-reduction service provision which is quite as expected.

The present study highlighted the need for future research utilizing qualitative methods to gain more detailed insight into the provision of DRP-reduction services. It is needed, also, to know more details about providers and non-providers of DRP-reduction services, to identify the characteristics of pharmacists providing enhanced services. To realize enhanced services, coaching pharmacists to provide DRP-reduction services is needed; utilizing a research design that pilots the launch of the service. In that process, redistribution of workflow can be sought enhancing the role of pharmacist assistant to deliver non-judgmental activities and keeping pharmacist input for core clinical, cognitive, and high-level services.

The present study has few limitations. The bias of social desirability (over-reporting of good behavior) [33], which is inherent in such types of surveys could be a limitation to this study. However, social desirability bias was, to a certain degree, controlled by the anonymous distribution of the survey and the careful formatting of questions minimizing leading questions that would lead to predictable answers. In addition, the present study addressed the self-rated provision of services; no measure of the actual provision nor the quality/significance of the service was used. The small sample size of pharmacists necessitates caution when interpreting the study results. Other issues could affect the generalizability of the results, such as most of the sample was young and females. Convenience sampling was used to recruit participants because it allows faster data collection from potentially available participants. Selection bias, on the other hand, is a drawback of convenience sampling, limiting the generalizability of the results. However, the high response rate (95.2%) achieved in our study may lessen any potential bias.

## Conclusion

Community pharmacists are in a strategic position to observe and correct DRPs. In this study, the overall provision of DRP and the utilization of reduction services was suboptimal in the community pharmacists surveyed. However, a number of services were delivered to a slightly higher degree, such as assessments, interventions, referrals, assessing for DRPs by asking patients, setting goals for DRP issues, providing interventions in the pharmacy, referring patients to medical doctors and/or other pharmacists, documenting DRPs, documenting interventions to resolve DRPs, and documenting plans to resolve DRPs. Whether pharmacists understand their proposed roles as healthcare providers extend beyond classical product-oriented activities needs further investigation. Curricula of undergraduate pharmacy degrees should emphasize the roles of community pharmacists and the nature of pharmaceutical care services anticipated in such settings. On the other hand, enhancing services provided through pharmacies require greater interaction between pharmacists and the public and should include health topics complementary to medication use, such as lifestyle and adherence issues. To maximize the role of pharmacists, perceived barriers must be analyzed and measures to overcome such barriers are necessary to enable pharmacists to perform the healthcare tasks and activities to an acceptable level.
